# A new phase of identifying RNA-binding proteins: Plant phase extraction

**DOI:** 10.1093/plcell/koad153

**Published:** 2023-05-30

**Authors:** Nora Flynn

**Affiliations:** Assistant Features Editor, The Plant Cell, American Society of Plant Biologists, USA; Department of Botany and Plant Sciences, University of California Riverside, Riverside, CA 92507, USA

From splicing to localization to degradation, RNA-binding proteins (RBPs) influence the destiny of an RNA and provide an important layer of posttranscriptional regulation. Historically, RBPs were identified by crosslinking (or bonding together) RNA and interacting proteins and isolating the RNA-protein complexes using oligo(dT)-containing beads (Ramanathan et al. 2019). Unfortunately, the use of oligo(dT) leads to an underestimation of RBPs because it only captures polyadenylated RNA, which is approximately 5% of cellular RNA.

**Figure 1.**
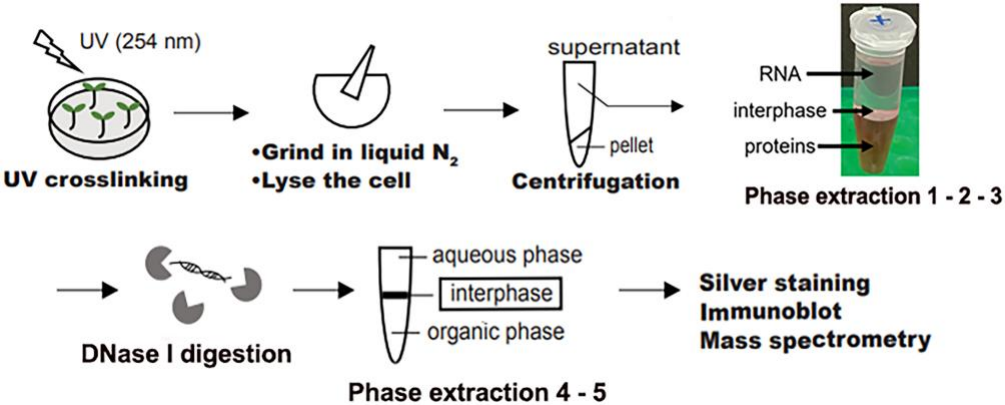
Summary of the steps of PPE to identify plant RBPs. Centrifugation yields RNA in an upper aqueous layer, proteins in a lower organic layer, and RNA-protein complexes in the interphase. The interphase layer is subjected to multiple rounds of centrifugation and phase extraction. Adapted from Zhang et al. (2023), Figure 1A and Supplemental Figure S1A.

To overcome this shortcoming, protocols to capture more diverse RNA-protein complexes were developed (Ramanathan et al. 2019). Phase separation is one method to isolate RNA-protein complexes without excluding nonpolyadenylated RNA. Strategies like orthogonal organic phase separation and protein-crosslinked RNA extraction take advantage of the intrinsic properties of RNA and proteins to centrifuge the molecules into a lower layer of proteins and an upper layer of RNA (Queiroz et al. 2019; Trendel et al. 2019). The cross-linked RNA-protein complexes share properties of both molecules and are collected from the middle layer, known as the interphase (Fig 1.).

Despite the promise of phase separation–based strategies, they have yielded mediocre results when applied to plant tissue. A trial of orthogonal organic phase separation using Arabidopsis tissue had low overlap to previous RBP datasets and even identified a non-RBP, histone H4, as an RBP candidate (Liu et al. 2020). Is plant tissue incompatible with phase-separation-based approaches?

In this issue, **Yong Zhang and colleagues** (Zhang et al. 2023) introduce a new strategy called plant phase extraction (PPE) that effectively identifies RBPs in plants using phase separation. PPE incorporates several major modifications to previous protocols, such as altered crosslinking conditions and increased rounds of phase extraction. During the rounds of phase extraction, the interphase layer is repeatedly collected and reseparated to reduce contaminants and obtain high-quality RNA-protein complexes (Fig. 1). Immunoblotting of the final PPE interphase validated the accuracy of the approach by showing that a known RBP, AGO1, could be detected after 5 rounds of phase separation, whereas a known non-RBP, histone H4, could not.

Once established, PPE followed by mass spectrometry was used to compare the RNA-binding proteome (RBPome) of Arabidopsis root or leaf tissue, with or without exposure to high-salt conditions. PPE could identify salt-responsive RBPs, the majority of which were tissue specific. One example of a salt-responsive RBP was ACTIN DEPOLYMERIZING FACTOR2, which had reduced RNA binding in the leaf but increased binding in the root when salt levels were high, demonstrating tissue-specific stress response.

A huge benefit of PPE is that it can collect RBPs associated with nonpolyadenylated RNA. By categorizing RBPs found by both PPE and previous oligo(dT)-based approaches as the “poly(A) RBPome,” the authors could separate out the RBPs found solely by PPE as the “non-poly(A) RBPome.” Nearly one-half of the RBPs found by PPE were not identified in previous oligo(dT)-based studies, affording a glance into RBPs that may associate with nonpolyadenylated RNA. Interestingly, RBPs from the non-poly(A) RBPome had differently enriched domains than RBPs from the poly(A) RBPome, highlighting the uniqueness of these 2 populations.

Finally, to verify the RNA binding of RBPs identified by PPE, the authors developed an RNA pull-down assay that used polyadenylated Arabidopsis RNA on oligo(dT) beads as bait to capture an input RBP. If the RBP binds to RNA and is pulled down on the beads, it can be visualized via immunoblotting. Using this technique, the binding capacity of several RBPs was confirmed.

PPE marks a great improvement in methodology to identify RBPs in plant tissue by phase separation. Although PPE was not able to identify all RBPs previously discovered by competing approaches, it still identified comparatively more RBPs than other methods. Additionally, a majority of these RBPs overlapped with other RNA-binding proteomes. Further exploring the RNA partners of some of the identified RBPs will provide deeper insight into their potential roles.
